# (*E*)-2-[1-(3-Chloro-4-fluoro­phen­yl)ethyl­idene]hydrazinecarbothio­amide

**DOI:** 10.1107/S1600536810051093

**Published:** 2010-12-11

**Authors:** Xiu-Rong Zhai

**Affiliations:** a Department of Chemisry and Chemical Engineering, Jining University, 273155 Qufu, Shandong, People’s Republic of China

## Abstract

In the crystal of the title compound, C_9_H_9_ClFN_3_S, the molecules are inter­connected by N—H⋯S and N—H⋯F hydrogen bonds. There are two different N—H⋯S hydrogen bond: the stronger one links mol­ecules into infinite chains along the *b* axis with graph-set motif *C*(4), while the weaker N—H⋯S hydrogen bond combines with the previous one into an *R*_2_^2^(8) network. Moreover, the chains are linked into layers parallel to (102) by weak N—H⋯F hydrogen bonds, which form an *R* ^2^_2_(22) ring motif. In addition, there are also weak π–π inter­actions between the benzene rings of adjacent mol­ecules [centroid–centroid distance = 3.8997 (15) Å].

## Related literature

For the chemistry and biological activity of thio­semi­carba­zones and their derivatives, see: Kasuga *et al.* (2001 ▶); Fonari *et al.* (2003 ▶); Amoedo *et al.* (2006 ▶); Mirsha *et al.* (2006 ▶); Kovala-Demertzi *et al.* 2007 ▶; Tarafder *et al.* (2008 ▶); Kizilcikli *et al.* (2004 ▶). For bond-length data, see: Allen *et al.* (1987 ▶). For graph-set theory, see: Etter *et al.* (1990 ▶).
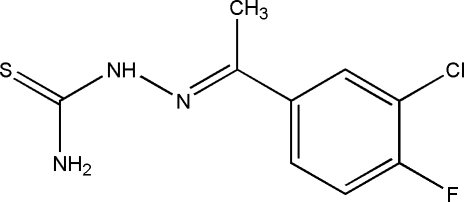


## Experimental

### 

#### Crystal data

C_9_H_9_ClFN_3_S
*M* *_r_* = 245.70
Monoclinic, 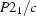

*a* = 7.8226 (10) Å
*b* = 8.2415 (12) Å
*c* = 18.4582 (19) Å
β = 112.244 (4)°
*V* = 1101.4 (2) Å^3^
*Z* = 4
Mo *K*α radiation
μ = 0.52 mm^−1^
*T* = 296 K
0.15 × 0.12 × 0.10 mm

#### Data collection

Bruker SMART APEXII CCD area-detector diffractometer
Absorption correction: multi-scan (*SADABS*; Bruker, 2005 ▶) *T*_min_ = 0.926, *T*_max_ = 0.950
6461 measured reflections
2379 independent reflections
2069 reflections with *I* > 2σ(*I*)
*R*_int_ = 0.018

#### Refinement

*R*[*F* ^2^ > 2σ(*F* ^2^)] = 0.044
*wR*(*F* ^2^) = 0.128
*S* = 1.06
2379 reflections
137 parameters
H-atom parameters constrained
Δρ_max_ = 0.47 e Å^−3^
Δρ_min_ = −0.47 e Å^−3^

### 

Data collection: *APEX2* (Bruker, 2005 ▶); cell refinement: *SAINT* (Bruker, 2005 ▶); data reduction: *SAINT*; program(s) used to solve structure: *SHELXTL* (Sheldrick, 2008 ▶); program(s) used to refine structure: *SHELXTL*; molecular graphics: *SHELXTL*; software used to prepare material for publication: *SHELXTL*.

## Supplementary Material

Crystal structure: contains datablocks global, I. DOI: 10.1107/S1600536810051093/fb2232sup1.cif

Structure factors: contains datablocks I. DOI: 10.1107/S1600536810051093/fb2232Isup2.hkl

Additional supplementary materials:  crystallographic information; 3D view; checkCIF report

## Figures and Tables

**Table 1:** Hydrogen-bond geometry (Å, °)

*D*—H⋯*A*	*D*—H	H⋯*A*	*D*⋯*A*	*D*—H⋯*A*
N1—H1*B*⋯S1^i^	0.86	2.50	3.327 (2)	161
N2—H2⋯S1^ii^	0.86	2.73	3.4817 (19)	147
N1—H1*A*⋯F1^iii^	0.86	2.30	3.051 (2)	146

Symmetry codes: (i) 
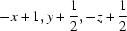
; (ii) 
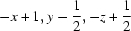
; (iii) 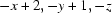.

**Fig. 1.**
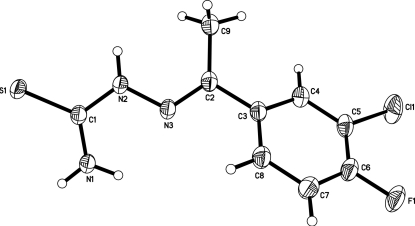
The title molecule with displacement ellipsoids drawn at the 30% probability level.

**Fig. 2.**
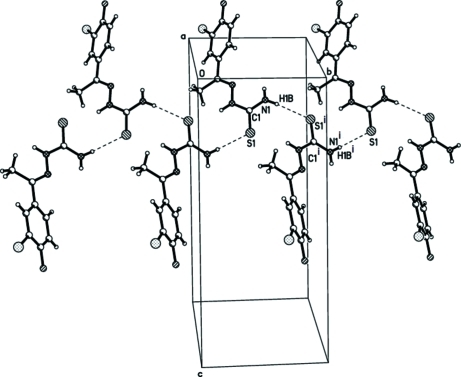
A view showing infinite chains with the graph-set motif C(4) corresponding to the C1-N1-H1B···S1i hydrogen bonds (dashed lines) [symmetry code i: 1-x, y+1/2, -z+1/2].

**Fig. 3.**
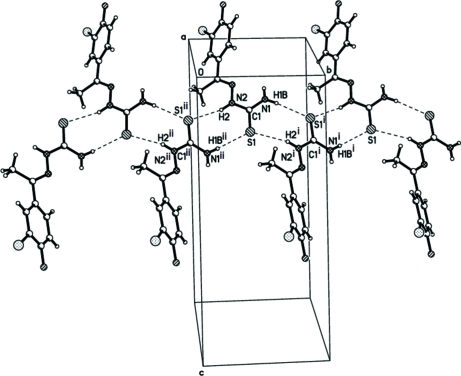
A view showing the network R22(8) pertinent to hydrogen bonds S1—C1—N2—H2···S1ii—C1ii—N1ii—H1Bii···S1 (the dashed lines) [symmetry code ii: 1-x, y-1/2, -z+1/2].

## supplementary crystallographic information

### Comment

Thiosemicarbazones constitute an important class of N, S donor ligands and have been investigated because of their chemistry and biological activities (Kasuga *et al.*, 2001; Fonari *et al.*, 2003; Kizilcikli *et al.*, 2004; Amoedo *et al.*, 2006; Mirsha *et al.*, 2006; Kovala-Demertzi *et al.*, 2007; Tarafder *et al.*, 2008.) In order to search for new thiosemicarbazones, the title compound has been synthesized and its crystal structure is reported here.

In the title molecule (Fig. 1), the bond lengths and angles are normal (Allen *et al.*, 1987). In the crystal structure, the molecules are linked by intermolecular N1—H1B···S1 hydrogen bonds, forming infinite chains with the graph-set motif *C*(4) (Tab. 1; Fig. 2; Etter *et al.*, 1990). The involved atoms in this graph set motif are S1—C1—N1—H1B···S1^i^ [the symmetry code i: 1 - *x*, 1/2 + *y*, 1/2 + *-z*]. Moreover, this interaction is strengthened by an almost parallel, a somewhat longer N2—H2···S1 hydrogen bond (Tab. 1; Fig. 3). Both these H bonds form a network *R*^2^_2_(8). (The involved atoms in the network are C1^i^—N1^i^—H1B^i^···S1^ii^—C1^ii^—N2^ii^—H2^ii^···S1^i^ [the symmetry code i: 1 - *x*, 1/2 + *y*, 1/2 - *z*; ii: 2 - *x*, 1 - *y*, -*z*]). The chains are further linked into the layers by weak N—H···F hydrogen bonds. The N—H···F hydrogen bonds form a ring motif *R*^2^_2_(22). [The involved atoms in the ring motif are N1—H1A···F1^ii^—C6^ii^—C5^ii^—C4^ii^—C3^ii^—C2^ii^—N3^ii^— N2^ii^—C1^ii^—N1^ii^—H1A^ii^···F1—C6—C5—C4—C3—C2—N3— N2—C1 (the symmetry code ii: 2 - *x*, 1 - *y*, -*z*)]. The layers are parallel to (1 0 2).

In addition, there are also present weak π-electron—π-electron interactions between the benzene rings of the adjacent molecules (the centroid—the centroid distance equals to 3.8997 (15) Å; the symmetry code: 2 - *x*, 1 - *y*, -*z*). Moreover, there is also even a weaker π-electron—π-electron ring interaction between another benzene ring from the other side with the centroid-centroid distance equal to 4.3962 (15)Å (the symmetry code: 2 - *x*, -*y*, -*z*).

### Experimental

The title compound was synthesized by the reaction of hydrazinecarbothioamide (1 mmol, 91.1 mg) with 1-(3-chloro-4-fluoro-phenyl)-ethanone (1 mmol, 172.6 mg) in anhydrous ethanol (20 ml) under reflux conditions (353 K) for 6 h. The solvent was removed under reduced pressure and the solid product has been recrystallized from 10 ml of anhydrous ethanol. The yield was 82%. After six days, colourless block-shaped crystals with approx. size 0.2 × 0.1 × 0.1 mm were obtained.

### Refinement

All the H atoms could be discerned in the difference electron density map. However, they were situated into the idealized positions and refined within the riding atom approximation. The used constraints: N_primary/secondary amine_—H_primary/secondary amine_=0.86; C_aryl_—H_aryl_ = 0.93; C_methyl_—H_methyl_ = 0.96 Å. *U*_iso_(H_primary/secondary amine/aryl_)=1.2 *U*_eq_(C_primary/secondary amine/aryl_); *U*_iso_(H_methyl_) =1.5*U*_eq_(C_methyl_). A rotating group model was used for the refinement of the positions of the methyl group. The minimal and the maximal residual electron densities were located at 0.85 and 0.70 Å from Cl1, respectively.

### Crystal data

**Table:**

C_9_H_9_ClFN_3_S	*F*(000) = 504
*M**_r_* = 245.70	*D*_x_ = 1.482 Mg m^−^^3^
Monoclinic, *P*2_1_/*c*	Mo *K*α radiation, λ = 0.71073 Å
Hall symbol: -P 2ybc	Cell parameters from 3779 reflections
*a* = 7.8226 (10) Å	θ = 2.4–28.2°
*b* = 8.2415 (12) Å	µ = 0.52 mm^−^^1^
*c* = 18.4582 (19) Å	*T* = 296 K
β = 112.244 (4)°	Block, colorless
*V* = 1101.4 (2) Å^3^	0.15 × 0.12 × 0.10 mm
*Z* = 4	

### Data collection

**Table:**

Bruker SMART APEXII CCD area-detector diffractometer	2379 independent reflections
Radiation source: fine-focus sealed tube	2069 reflections with *I* > 2σ(*I*)
graphite	*R*_int_ = 0.018
φ and ω scans	θ_max_ = 27.0°, θ_min_ = 2.4°
Absorption correction: multi-scan (*SADABS*; Bruker, 2005)	*h* = −9→5
*T*_min_ = 0.926, *T*_max_ = 0.950	*k* = −9→10
6461 measured reflections	*l* = −20→23

### Refinement

**Table:**

Refinement on *F*^2^	Primary atom site location: structure-invariant direct methods
Least-squares matrix: full	Secondary atom site location: difference Fourier map
*R*[*F*^2^ > 2σ(*F*^2^)] = 0.044	Hydrogen site location: difference Fourier map
*wR*(*F*^2^) = 0.128	H-atom parameters constrained
*S* = 1.06	*w* = 1/[σ^2^(*F*_o_^2^) + (0.0639*P*)^2^ + 0.6228*P*] where *P* = (*F*_o_^2^ + 2*F*_c_^2^)/3
2379 reflections	(Δ/σ)_max_ < 0.001
137 parameters	Δρ_max_ = 0.47 e Å^−^^3^
0 restraints	Δρ_min_ = −0.47 e Å^−^^3^

### Special details

**Table:**

Geometry. All e.s.d.'s (except the e.s.d. in the dihedral angle between two l.s. planes) are estimated using the full covariance matrix. The cell e.s.d.'s are taken into account individually in the estimation of e.s.d.'s in distances, angles and torsion angles; correlations between e.s.d.'s in cell parameters are only used when they are defined by crystal symmetry. An approximate (isotropic) treatment of cell e.s.d.'s is used for estimating e.s.d.'s involving l.s. planes.
Refinement. Refinement of *F*^2^ against ALL reflections. The weighted *R*-factor *wR* and goodness of fit *S* are based on *F*^2^, conventional *R*-factors *R* are based on *F*, with *F* set to zero for negative *F*^2^. The threshold expression of *F*^2^ > σ(*F*^2^) is used only for calculating *R*-factors(gt) *etc*. and is not relevant to the choice of reflections for refinement. *R*-factors based on *F*^2^ are statistically about twice as large as those based on *F*, and *R*- factors based on ALL data will be even larger.

### Fractional atomic coordinates and isotropic or equivalent isotropic displacement parameters (Å^2^)

**Table:**

	*x*	*y*	*z*	*U*_iso_*/*U*_eq_	
Cl1	1.35789 (9)	0.14464 (12)	0.01370 (4)	0.0767 (3)	
S1	0.48566 (10)	0.45517 (7)	0.27045 (4)	0.0579 (2)	
F1	1.0826 (2)	0.3057 (2)	−0.11859 (9)	0.0675 (4)	
N3	0.7524 (2)	0.3397 (2)	0.13785 (10)	0.0416 (4)	
N2	0.6771 (3)	0.3277 (2)	0.19401 (10)	0.0412 (4)	
H2	0.6824	0.2389	0.2192	0.049*	
N1	0.6073 (3)	0.5950 (2)	0.17163 (12)	0.0524 (5)	
H1A	0.6662	0.5951	0.1406	0.063*	
H1B	0.5564	0.6827	0.1790	0.063*	
C3	0.9182 (3)	0.2442 (2)	0.06572 (12)	0.0399 (4)	
C2	0.8562 (3)	0.2247 (2)	0.13192 (12)	0.0392 (4)	
C8	0.8048 (3)	0.3213 (3)	−0.00227 (13)	0.0484 (5)	
H8	0.6901	0.3597	−0.0059	0.058*	
C7	0.8590 (4)	0.3422 (3)	−0.06482 (14)	0.0525 (6)	
H7	0.7820	0.3930	−0.1105	0.063*	
C6	1.0288 (3)	0.2858 (3)	−0.05752 (13)	0.0476 (5)	
C5	1.1441 (3)	0.2103 (3)	0.00803 (14)	0.0462 (5)	
C4	1.0893 (3)	0.1874 (3)	0.07075 (13)	0.0441 (5)	
H4	1.1667	0.1345	0.1157	0.053*	
C1	0.5950 (3)	0.4612 (2)	0.20768 (12)	0.0394 (4)	
C9	0.9087 (4)	0.0788 (3)	0.18293 (15)	0.0576 (6)	
H9A	0.9228	0.1080	0.2352	0.086*	
H9B	1.0232	0.0362	0.1832	0.086*	
H9C	0.8139	−0.0020	0.1632	0.086*	

### Atomic displacement parameters (Å^2^)

**Table:**

	*U*^11^	*U*^22^	*U*^33^	*U*^12^	*U*^13^	*U*^23^
Cl1	0.0482 (4)	0.1172 (7)	0.0786 (5)	0.0102 (3)	0.0398 (3)	−0.0058 (4)
S1	0.0888 (5)	0.0384 (3)	0.0771 (4)	0.0073 (3)	0.0662 (4)	0.0041 (2)
F1	0.0798 (10)	0.0817 (10)	0.0629 (9)	−0.0066 (8)	0.0519 (8)	0.0007 (8)
N3	0.0465 (10)	0.0413 (9)	0.0478 (9)	0.0002 (7)	0.0301 (8)	−0.0025 (7)
N2	0.0518 (10)	0.0363 (8)	0.0485 (9)	0.0031 (7)	0.0336 (8)	0.0005 (7)
N1	0.0665 (12)	0.0402 (9)	0.0728 (13)	0.0084 (9)	0.0515 (11)	0.0074 (9)
C3	0.0403 (10)	0.0401 (10)	0.0458 (10)	−0.0019 (8)	0.0235 (9)	−0.0053 (8)
C2	0.0367 (10)	0.0430 (10)	0.0430 (10)	−0.0013 (8)	0.0210 (8)	−0.0059 (8)
C8	0.0451 (11)	0.0575 (13)	0.0495 (12)	0.0048 (10)	0.0260 (10)	0.0009 (10)
C7	0.0553 (13)	0.0600 (14)	0.0465 (12)	0.0028 (11)	0.0244 (10)	0.0039 (10)
C6	0.0557 (13)	0.0503 (12)	0.0493 (12)	−0.0102 (10)	0.0340 (10)	−0.0075 (9)
C5	0.0403 (11)	0.0521 (12)	0.0551 (12)	−0.0047 (9)	0.0282 (10)	−0.0112 (10)
C4	0.0407 (11)	0.0513 (12)	0.0455 (11)	0.0016 (9)	0.0220 (9)	−0.0036 (9)
C1	0.0415 (10)	0.0389 (10)	0.0457 (10)	0.0004 (8)	0.0255 (9)	−0.0017 (8)
C9	0.0665 (15)	0.0605 (14)	0.0584 (14)	0.0217 (12)	0.0379 (12)	0.0113 (11)

### Geometric parameters (Å, °)

**Table:**

Cl1—C5	1.723 (2)	C3—C2	1.484 (3)
S1—C1	1.681 (2)	C2—C9	1.486 (3)
F1—C6	1.354 (2)	C8—C7	1.383 (3)
N3—C2	1.279 (3)	C8—H8	0.9300
N3—N2	1.376 (2)	C7—C6	1.365 (3)
N2—C1	1.345 (2)	C7—H7	0.9300
N2—H2	0.8600	C6—C5	1.356 (3)
N1—C1	1.310 (3)	C5—C4	1.391 (3)
N1—H1A	0.8600	C4—H4	0.9300
N1—H1B	0.8600	C9—H9A	0.9600
C3—C8	1.386 (3)	C9—H9B	0.9600
C3—C4	1.387 (3)	C9—H9C	0.9600
			
C2—N3—N2	118.55 (17)	F1—C6—C5	119.1 (2)
C1—N2—N3	116.98 (16)	F1—C6—C7	118.3 (2)
C1—N2—H2	121.5	C5—C6—C7	122.5 (2)
N3—N2—H2	121.5	C6—C5—C4	119.6 (2)
C1—N1—H1A	120.0	C6—C5—Cl1	120.06 (17)
C1—N1—H1B	120.0	C4—C5—Cl1	120.32 (18)
H1A—N1—H1B	120.0	C3—C4—C5	119.5 (2)
C8—C3—C4	119.05 (19)	C3—C4—H4	120.3
C8—C3—C2	119.89 (18)	C5—C4—H4	120.3
C4—C3—C2	121.06 (19)	N1—C1—N2	117.44 (18)
N3—C2—C3	113.97 (18)	N1—C1—S1	121.95 (15)
N3—C2—C9	125.24 (19)	N2—C1—S1	120.60 (15)
C3—C2—C9	120.74 (17)	C2—C9—H9A	109.5
C7—C8—C3	121.3 (2)	C2—C9—H9B	109.5
C7—C8—H8	119.3	H9A—C9—H9B	109.5
C3—C8—H8	119.3	C2—C9—H9C	109.5
C6—C7—C8	118.0 (2)	H9A—C9—H9C	109.5
C6—C7—H7	121.0	H9B—C9—H9C	109.5
C8—C7—H7	121.0		
			
C2—N3—N2—C1	169.12 (19)	C8—C7—C6—C5	0.4 (4)
N2—N3—C2—C3	175.60 (17)	F1—C6—C5—C4	−179.2 (2)
N2—N3—C2—C9	−2.1 (3)	C7—C6—C5—C4	0.3 (4)
C8—C3—C2—N3	−31.7 (3)	F1—C6—C5—Cl1	1.3 (3)
C4—C3—C2—N3	147.8 (2)	C7—C6—C5—Cl1	−179.13 (19)
C8—C3—C2—C9	146.0 (2)	C8—C3—C4—C5	0.6 (3)
C4—C3—C2—C9	−34.4 (3)	C2—C3—C4—C5	−178.9 (2)
C4—C3—C8—C7	0.1 (3)	C6—C5—C4—C3	−0.8 (3)
C2—C3—C8—C7	179.6 (2)	Cl1—C5—C4—C3	178.62 (17)
C3—C8—C7—C6	−0.6 (4)	N3—N2—C1—N1	−5.8 (3)
C8—C7—C6—F1	179.9 (2)	N3—N2—C1—S1	175.61 (15)

### Hydrogen-bond geometry (Å, °)

**Table:**

*D*—H···*A*	*D*—H	H···*A*	*D*···*A*	*D*—H···*A*
N1—H1B···S1^i^	0.86	2.50	3.327 (2)	161
N2—H2···S1^ii^	0.86	2.73	3.4817 (19)	147
N1—H1A···F1^iii^	0.86	2.30	3.051 (2)	146

Symmetry codes: (i) −*x*+1, *y*+1/2, −*z*+1/2; (ii) −*x*+1, *y*−1/2, −*z*+1/2; (iii) −*x*+2, −*y*+1, −*z*.
